# European disaster management in response to the COVID-19 pandemic

**DOI:** 10.1007/s11299-020-00252-2

**Published:** 2020-09-08

**Authors:** Christian Wankmüller

**Affiliations:** Department of Operations, Energy, and Environmental Management, Universitaet Klagenfurt, Universitätsstraße 65 - 67, 9020 Klagenfurt, Austria

**Keywords:** COVID-19, Coronavirus, European disaster management, Disaster preparedness, Immediate response

## Abstract

Top priority of governments in containing the COVID-19 pandemic is “flattening the curve” which implies a slowing down of the virus’ spread across the entire population. The situation which European policymakers are facing at the moment is completely new and only few of them have the required experience to handle a disaster of such magnitude. What is important now is to avoid problems that repeatedly occurred in past disaster responses by learning the lessons and acting accordingly. This paper reflects on European disaster management in containing the spread of COVID-19 and uncovers response inefficiencies that are still present.

## Introduction

Disasters of recent years have caused thousands of victims and long-term ecological, social and economic damage to the affected areas. The Indian Ocean Tsunami in 2004, the Horn of Africa malnutrition crisis in 2011 and the Nepal Earthquake in 2015 are examples of devastating disasters, which put the resilience of society to the test. Since December 2019, the world population is once again facing a disaster of unexpected magnitude caused by a newly discovered coronavirus (SARS-CoV2). This virus leads to a severe acute respiratory infection (COVID-19), comparable to pneumonia, characterized by fever, cough and shortness of breath (Liu et al. 2020). This virus is remarkably dangerous, due to its ease of person-to-person transmission even by infected individuals who do not have any perceivable symptoms (Bai et al. 2020). This novel virus first attracted increased international attention in December 2019, when the Chinese government announced the first cases of infection in Wuhan, the capital city of Hubei, China. Since then, the virus has spread globally, resulting in the ongoing coronavirus pandemic. According to the Center for Systems Science and Engineering at Johns Hopkins University, a total number of 1,850,966 confirmed cases with 114,290 deaths in 185 countries worldwide had been reported by April 16th 2020 (Johns Hopkins University 2020). It is assumed that the number of unrecorded cases is considerably higher due to inconsistent testing and the non-availability of testing equipment in certain parts of the world. During the first weeks of the outbreak it quickly turned out that COVID-19 is much more dangerous than the conventional flu and therefore systematic interventions to contain the epidemic are urgently needed.

At this point, problems and inefficiencies experienced in recent mega disasters have to be avoided in order to efficiently contain the virus’ spreading across Europe. This paper sheds light on the lessons learned by European disaster management in response to COVID-19 and asks for major drawbacks that still occur. The article continues with a short introduction to disaster management and observed inefficiencies of past disaster response (Sect. 2). Section 3 then reflects on the lessons learned by European governments and discusses alternative approaches to handle the situation. Section 4 concludes the paper with final remarks related to the topic.

## Disasters, learnings and the role of academia

As with the response to other extreme disasters—naturally born or man-made—the alleviation of COVID-19 victims’ suffering has top priority and constitutes the main objective of ongoing containment measures taken by local and global governments. In order to provide beneficiaries with critical emergency items, such as masks, oxygen and personal protective equipment, the need to implement and maintain an effective and efficient disaster management is evident. Disaster management which combines all measures and processes to prepare for and respond to disasters has been recognized as the key to successful disaster relief (Coppola 2006). From a scientific point of view, much qualitative and quantitative research has been conducted in recent years to provide policymakers and stakeholders with recommendations to design highly efficient disaster management strategies (Kovács et al. 2019). In this regard, research activities have been focusing on the four stages of the disaster management cycle that categorizes disaster management into four phases—mitigation, preparedness, response and recovery (Cozzolino 2012). Here, special attention is put on disaster preparedness and immediate response phases, due the direct impact of activities, programs and measures generated for coping with disasters.

However, the increase in research has not led to a proportional impact on disaster management practice (Donahue and Tuohy 2006; Kunz et al. 2017). There are multiple obstacles and challenges that prevent appropriate exchange of disaster-related information and know-how. On the one hand, it’s the researchers’ low level of practical experience that hampers their understanding of what really matters in disaster management. On the other hand, disaster managers are often rather skeptical towards the practical implementability of generated research results and therefore back off from realizing certain performance enabler. Especially the work by Donahue and Tuohy (2006) reveals that the lessons learned from past disasters are quite limited and organizations’ readiness to better prepare for future incidents is relatively low in the midst of, and immediate following a disaster. Using an exploratory research approach, the authors analyzed official reports and processed expert interviews following major disasters such as Hurricane Katrina (2005), the September 11th attacks (2001), Columbia space shuttle crash (2003) and others. The authors found out that in major response missions multiple communication problems arose, supplies were lost, situational awareness broke down, making it difficult to deliver response capabilities to the people and places in need. Almost all disaster missions where characterized by uncoordinated leadership, failed communications, weak planning, resource constraints and poor public relations that result in observed deficiencies. Given the time pressure and highly uncertain environment in which practitioners have to operate and take far-reaching decisions, their behavior is understandable to a certain extent.

## Lessons (not) learned by European disaster management

When following the media updates on current reactions of European governments in answer to COVID-19, one could argue that the lessons learned from past disasters (e.g. the migration crisis in 2015) are fairly meager and problems in disaster management have not improved over the last few years. First and foremost, there is the tendency of European countries to perceive disaster risk and to evaluate disaster impacts in very different ways. While some governments did not perceive COVID-19 as a threat to citizens, health systems and general living, others recognized the acute need for action at a very early stage. Accompanied by high levels of uncertainty regarding the virus’ spread, this cocktail led to uncoordinated domestic implementations of measures to manage the pandemic. Not only the speed of reaction but also the mix of measures differed extremely from one country to another. For instance, a complete lockdown (i.e. all events suspended, schools closed, non-essential shops closed, non-essential movement banned and land borders closed) before the third death caused by COVID-19 was performed by Greece, Poland, Austria, Portugal, Hungary and the Czech Republic. Other countries including France, Spain, the United Kingdom or Germany were more reluctant to direct the public to adhere to recommendations regarding personal hygiene, quarantine, travel restrictions, or the closure of public buildings (Hale et al. 2020). Those countries introduced restrictive measures with massive time delays ranging between 2 and 15 days after the respective country’s third death was recorded. At this point, there is an urgent question as to why a European-wide coordination in terms of timing and implementing of COVID-19 strategies is/was not pursued by the member states. Isolated decision-making in disaster situations, as observed here, generally leads to less performance and lower efficiency levels of measures taken. There is a lot of evidence about the negative consequences of such coordination failures as for instance in the large-scale responses in the 2004 Indian Ocean Tsunami, following Hurricane Katrina in 2005 or after the 2010 Haitian Earthquake (Costa et al. 2015). Instead, the active sharing of disaster-related information and distributed decision-making (i.e. group decision-making) is recommended here (Stephenson 2006). The joint integration of individual view-points, knowledge and expertise of single members within this kind of coordination network enhances the outcome of the decision-making process, as the perception of fairness, acceptance of the decisions made and identification of the group with decision impacts are increased (Brodbeck et al. 2007). This kind of coordination mechanism would be appropriate and even necessary to jointly master the COVID-19 crisis.

Other recurring problems are related to insufficient communication and the inconsistent handling of resource constraints. Aside from encouraging physical (or social) distancing to slow the rate of transmission, more and more governments are instructing their citizens to wear masks and to use disinfectant spray in supermarkets and even public places. With more than 446 million^1^ people living in the entire European Union, a massive imbalance between demand and supply in this regard is almost inevitable. In order to relax critical supply shortages, some countries like Austria and France have called for international support and built an air bridge to China and imported tons of protective equipment (Austria Press Agency 2020; Metropole 2020). However, inter-European cooperation and communication about potential resource constraints has not had top priority. For instance, Germany issued an export ban on masks that were initially dedicated for the Austrian market without reasonable grounds. Also, Switzerland was impacted by the lack of German cooperation, causing turbulences in the internal supply chains for masks and other protective items (Bloomberg 2020). Only after interventions by the European Commission did Germany lift the ban and agree to releasing exports to neighboring countries (Reuters 2020). In this context, European disaster management and involved governments have/had problems to operationalize common strategies proposed by the humanitarian logistics literature to pool resources and to share them jointly (Balcik et al. 2010; Naor et al. 2018).

## Concluding remarks

Probably the aforementioned inefficiencies could have been avoided by more efficient preparedness activities on a European level. For years, academia has highlighted the relevance and importance of disaster preparedness for a targeted and adequate response after disasters hit (Hale and Moberg 2005; Kunz et al. 2014). Having in mind the fact that experts have consistently been warning that the next global pandemic is not a matter of if but of when, the preparedness activities of the European countries against the COVID-19 pandemic were relatively scant (Gates 2015). For sure, governments have corresponding plans to hand, but the vague (arbitrary) actions set by certain European countries imply low levels of comprehensive preparedness and weak planning. Actually, policymakers would have had enough time to prepare and learn from Asian countries but the geographic dispersion may have understated the situation’s criticality. Unfortunately, the negative consequences of the above outlined problems became a sad reality in some European countries through collapsed health systems and thousands of deaths. On conclusion, it is high time to learn the lessons, to better prepare for future disasters and to jointly respond to COVID-19 according to the official motto of the European Union “in varietate concordia”.

## Footnotes

1 Eurostat, Population change—Demographic balance and crude rates at national level, retrieved from https://appsso.eurostat.ec.europa.eu/nui/show.do?dataset=demo_gind&lang=en [accessed on April 15th 2020].
